# Negative Pregnancy Experiences and Psychological Distress: Associations with Active Coping and Marital Satisfaction

**DOI:** 10.3390/medicina62081512

**Published:** 2026-08-06

**Authors:** Elena Otilia Vladislav, Corina Ioana Paica-Stoian, Diana Antonia Iordăchescu, Anca Maria Panaitescu, Claudia Mehedințu, Nicolae Gică

**Affiliations:** 1 Department of Applied Psychology and Psychotherapy, Faculty of Psychology and Educational Sciences, University of Bucharest, 050663 București, Romania; elena.vladislav@fpse.unibuc.ro; 2 Filantropia Clinical Hospital of Obstetrics and Gynecology, 011132 București, Romania; diana.antonia.iordachescu@gmail.com (D.A.I.); anca.panaitescu@umfcd.ro (A.M.P.); claudia.mehedintu@umfcd.ro (C.M.); gica.nicolae@umfcd.ro (N.G.); 3 Clinical Department of Obstetrics and Gynecology, “Carol Davila” University of Medicine and Pharmacy, 050474 București, Romania

**Keywords:** high-risk pregnancy, prenatal negative experiences, coping strategies, marital satisfaction

## Abstract

*Background and Objectives*: High-risk pregnancy increases vulnerability to psychological distress, but protective mechanisms remain poorly understood. This study compared emotional distress and coping strategies between women with high- and low-risk pregnancies and investigated whether active coping and marital satisfaction moderated associations between negative pregnancy experiences, prenatal depression and perceived stress. *Materials and Methods*: A cross-sectional study was conducted among 195 pregnant women aged 18–51 years (M = 31.76, SD = 5.32), including 102 women with high-risk pregnancies and 93 women with low-risk pregnancies. Participants completed the EPDS, STAI, PSS-10, PRAS, PES, COPE, and CSI. Independent-samples t tests, correlation analyses, and moderation analyses were conducted. *Results*: Women with high-risk pregnancies reported significantly higher levels of prenatal depression (*p* = 0.032) and pregnancy-related anxiety (*p* < 0.001) than women with low-risk pregnancies. They also reported lower socio-emotional support (*p* = 0.036) and mental disengagement coping (*p* = 0.022). Negative pregnancy experiences were positively associated with state anxiety, pregnancy-related anxiety, prenatal depression, and perceived stress after controlling for pregnancy risk status (all *p* < 0.001). Maladaptive coping was generally associated with higher emotional distress, whereas adaptive coping, particularly positive reinterpretation and planning, was associated with lower levels of prenatal depression and anxiety. Active coping moderated the association between negative pregnancy experiences and perceived stress (β = −0.16, *p* = 0.016). Marital satisfaction moderated the association between prenatal depression and perceived stress among women with high-risk pregnancies (β = −0.21, *p* = 0.016). *Conclusions*: Active coping and marital satisfaction were associated with lower psychological distress and may represent potentially modifiable psychological resources for women experiencing high-risk pregnancies. Although the cross-sectional design precludes causal inferences, these findings highlight the potential value of integrating psychological screening, coping-focused interventions, and partner involvement into prenatal care, particularly for women with high-risk pregnancies.

## 1. Introduction

Pregnancy represents a period of substantial psychological reorganization characterized by increased vulnerability to stress, anxiety and depressive symptoms, even under physiological conditions [1,2,3]. Contemporary biopsychosocial models conceptualize the prenatal period as a dynamic process of emotional and cognitive adaptation in which the individual’s appraisal of events and the availability of internal and external resources play a central role in shaping psychological outcomes. Within this framework, the transactional theory of stress and coping offers a relevant explanatory perspective, suggesting that the psychological impact of pregnancy is mediated by subjective appraisal processes and the coping strategies mobilized in response to perceived demands [4,5].

High-risk pregnancy constitutes a particularly salient stressor, significantly amplifying psychological vulnerability. Empirical evidence consistently indicates higher levels of anxiety, depression, perceived stress and overall psychological distress among women with high-risk pregnancies compared to those with uncomplicated pregnancies [5,6,7,8,9]. Furthermore, psychological distress during pregnancy has been associated with poorer health-related quality of life and reduced maternal well-being, highlighting the importance of routine psychological assessment during prenatal care [9].

Systematic reviews further confirm that the majority of studies report decreased well-being and increased psychological symptomatology in high-risk contexts [10,11]. From a phenomenological perspective, women often describe persistent fears related to pregnancy outcomes, a heightened sense of uncertainty and loss of control, as well as feelings of guilt and social isolation [12,13]. Moreover, medicalization processes such as hospitalization or the assignment of a “high-risk” label may be experienced as psychologically traumatic, further intensifying emotional distress. Recent findings from the COVID-19 pandemic context have further underscored the fragility of this population, documenting elevated levels of anxiety, depression and stress [14,15].

Despite this heightened vulnerability, high-risk pregnancy also mobilizes a wide range of coping strategies, which may function adaptively or maladaptively. Adaptive coping strategies—such as problem-focused coping, positive reappraisal and positive spiritual coping—have been consistently associated with lower levels of distress and better psychological adjustment [11,16,17]. In contrast, avoidant or passive coping strategies are linked to higher levels of anxiety and depression [18], while negative forms of spiritual coping may exacerbate depressive symptomatology, particularly in high-risk contexts [19,20]. Comparative findings further suggest that women with high-risk pregnancies tend to rely less on problem-focused strategies and more on avoidance or rigid emotional control than women with low-risk pregnancies [6,18].

Although the literature consistently documents elevated distress levels in women with high-risk pregnancies and highlights the general role of coping processes, several important limitations remain. First, many studies rely primarily on global indicators of anxiety and depression, without incorporating pregnancy-specific dimensions such as pregnancy-related anxiety or subjective prenatal experiences. Second, the relationships between negative pregnancy experiences and multiple indicators of emotional distress have been insufficiently examined within integrated models, particularly in samples of women with high-risk pregnancies. Third, although coping has been widely investigated, there is a need to clarify the differentiated roles of adaptive and maladaptive strategies in relation to prenatal anxiety and depression beyond descriptive or unidimensional approaches. In addition, direct comparative studies between high-risk and low-risk pregnancies using integrative explanatory frameworks remain relatively limited.

Against this background, the present study aims to contribute to the existing literature by adopting an integrative approach to the relationship between emotional vulnerability and coping strategies during pregnancy.

Specifically, the objectives of the study are:

1. To compare prenatal depression, pregnancy-related anxiety, and coping strategies between women with high-risk and low-risk pregnancies.
2. To examine whether negative pregnancy experiences are associated with emotional distress independently of pregnancy risk status.
3. To investigate the associations between adaptive and maladaptive coping strategies and prenatal emotional distress.
4. To examine whether active coping moderates the association between negative pregnancy experiences and perceived stress.
5. To examine whether marital satisfaction moderates the association between prenatal depression and perceived stress among women with high-risk pregnancies.

In line with these objectives, the following hypotheses were formulated:

• **H1a.** Women with high-risk pregnancies will report higher levels of prenatal depression and pregnancy-related anxiety than women with low-risk pregnancies.
• **H1b.** Women with high-risk pregnancies will report lower use of socio-emotional support and mental disengagement coping strategies than women with low-risk pregnancies.
• **H2.** Negative pregnancy experiences will be positively associated with perceived stress, state anxiety, pregnancy-related anxiety and prenatal depression.
• **H3.** Maladaptive coping strategies will be positively associated with prenatal anxiety and depression, whereas adaptive coping strategies will be negatively associated with these indicators of emotional distress.
• **H4.** Active coping will moderate the positive association between negative pregnancy experiences and perceived stress.
• **H5.** Marital satisfaction will moderate the positive association between prenatal depression and perceived stress among women with high-risk pregnancies.

Overall, the present study advances the literature by integrating pregnancy-specific emotional processes, subjective experiences and coping mechanisms within a unified explanatory framework of prenatal adjustment. This approach provides a more nuanced understanding of emotional vulnerability in high-risk pregnancies and has direct implications for the development of targeted psychological interventions aimed at supporting maternal mental health.

## 2. Materials and Methods

### 2.1. Population

The study population consisted of 195 pregnant women recruited from the Clinical Hospital Filantropia, Bucharest. Their ages ranged from 18 to 51 years (M = 31.76; SD = 5.32). Most participants were married (87.2%, *n* = 170), and 10.8% (*n* = 21) reported living with a partner. The other categories (divorced, widowed, separated or other situations) were poorly represented in the study sample. Regarding the environment of origin, 74.4% of the participants (*n* = 145) came from urban areas, and 25.6% (*n* = 50) came from rural areas.

The level of education of the participants indicates a predominance of higher education, the majority of them having a bachelor’s degree (40%) or postgraduate studies (39%). The other educational levels (high school and sub-university or incomplete university education) were represented in a significantly lower proportion.

Regarding professional status, the majority of participants were employed (85.6%, *n* = 167), while lower percentages were recorded for the housewife (7.7%, *n* = 15) and self-employed (6.7%, *n* = 13) categories, as shown in Table 1a.

In terms of economic status, most participants fell into the middle income category (79.5%, *n* = 155), followed by those with high income (12.8%, *n* = 25) and low income (5.1%, *n* = 10), with a small percentage preferring not to respond (2.6%, *n* = 5).

Regarding maternity experience, 62.1% of participants (*n* = 121) were in their first pregnancy, and 37.9% (*n* = 74) had at least one previous pregnancy. In terms of pregnancy trimester, 47.7% of participants (*n* = 93) were in the third trimester, 36.9% (*n* = 72) in the second trimester, and 15.4% (*n* = 30) in the first trimester.

Concerning the risk status of the current pregnancy, 47.7% of participants (*n* = 93) were classified without medical risk pregnancies, while 52.3% (*n* = 102) had pregnancies with medical risk. Within the high-risk pregnancy group, the most prevalent medical conditions were Rh incompatibility (17.6%) and conception through assisted reproductive technologies (15.6%). Additional risk factors included preeclampsia and uterine pathology (11.7% each), gestational diabetes (9.9%), infectious diseases (7.9%), chronic maternal conditions and overweight status (6.9% each), and anemia (4.9%). Genetic abnormalities (4.0%), placenta previa (2.0%), and intrahepatic cholestasis of pregnancy (0.9%) were less commonly reported, as shown in Table 1b.

The clinical criterion for operationalizing the concept of high-risk pregnancy was developed by the co-authors of this study, as is evident from previously published studies [21,22,23,24].

An a priori power analysis using G*Power Program (Version 3.1.9.7) [25] indicated that a minimum sample of 176 participants (88 per group) was required to detect a medium effect size with 90% power at α = 0.05. The final sample of 195 participants exceeded this requirement. G*Power remains the standard tool for ensuring reproducible sample estimation. Recent studies [26,27] highlight its continued integration within open-science workflows to combat low statistical power and lower false-positive risks in behavioral frameworks.

### 2.2. Measures

Participants completed a series of seven self-report questionnaires administered either in person at the Filantropia Clinical Hospital of Obstetrics and Gynecology or online via Google Forms. The hospital is affiliated with “Carol Davila” University of Medicine and Pharmacy of Bucharest and is a tertiary care center serving both urban and rural populations in Romania, thus providing access to a diverse patient pool. Inclusion criteria were being pregnant, aged 18 years or older and able to read and understand Romanian.

#### 2.2.1. Edinburgh Postnatal Depression Scale (EPDS)

Prenatal depressive symptoms were assessed using the Edinburgh Postnatal Depression Scale (EPDS) [28]. The EPDS is a 10-item self-report questionnaire designed to assess depressive symptomatology during the perinatal period. Items are rated on a 4-point Likert scale, and total scores range from 0 to 30, with higher scores indicating greater depressive symptomatology. A cut-off score of 13 or higher was used to identify women at increased risk for clinically significant depression. A current prospective study demonstrated the current clinical utility of the EPDS as a reference tool in screening for perinatal depression through direct correlation with clinical interviews [29]. In the present study, internal consistency was good (α = 0.87).

#### 2.2.2. State–Trait Anxiety Inventory (STAI)

State anxiety was assessed using the State–Trait Anxiety Inventory (STAI) [30]. The STAI is a widely used measure of anxiety consisting of separate state and trait anxiety scales. A current longitudinal study used the STAI at weeks 12, 24, and 36 of pregnancy to map trajectories of gestational anxiety and its impact on infant neurodevelopment [31]. The present study employed the 20-item State Anxiety subscale, which assesses current anxiety symptoms. Items are rated on a 4-point Likert scale, and total scores range from 20 to 80, with higher scores indicating greater anxiety. Scores of 40 or above are generally considered indicative of clinically relevant anxiety symptoms. Internal consistency in the present sample was excellent (α = 0.95).

#### 2.2.3. Pregnancy Experiences Scale (PES)

Pregnancy-related experiences were assessed using the Pregnancy Experiences Scale (PES) [32]. The PES evaluates both positive pregnancy experiences (uplifts) and negative pregnancy experiences (hassles), providing an assessment of women’s subjective appraisal of pregnancy. An MRI study used the PES variant administered between weeks 26 and 36 of gestation to measure the correlation between maternal perceptions and infant brain connectivity [33]. Internal consistency was good for the positive experiences subscale (α = 0.79), very good for the negative experiences subscale (α = 0.85) and acceptable for the total score (α = 0.77).

#### 2.2.4. Perceived Stress Scale (PSS-10)

Perceived stress was measured using the 10-item Perceived Stress Scale (PSS-10) [34]. Participants rate the frequency of stress-related experiences during the previous month on a 5-point Likert scale ranging from 0 (“never”) to 4 (“very often”). Total scores range from 0 to 40, with higher scores indicating greater perceived stress. The scale includes two dimensions: perceived helplessness and perceived self-efficacy. Souza et al. [35] analyzed the sensitivity of the PSS-10 short version in modern obstetric populations to assess the emotional resilience of pregnant women. Internal consistency in the present study was acceptable (α = 0.71).

#### 2.2.5. Pregnancy-Related Anxiety Scale (PRAS)

Pregnancy-specific anxiety was assessed using the Pregnancy-Related Anxiety Scale (PRAS) [36]. The PRAS measures concerns related to childbirth, fetal health and obstetric complications. Items are rated on a 4-point Likert scale ranging from 1 (“not at all”) to 4 (“very much”), with higher scores indicating greater pregnancy-related anxiety. The study by Qannita et al. [37] highlighted the continuous predictive value of the PRAS scale to differentiate clinical fears focused strictly on the birth process from generalized anxiety. Internal consistency for our study was good (α = 0.83).

#### 2.2.6. Coping Orientation to Problems Experienced (COPE)

Coping strategies were assessed using the Coping Orientation to Problems Experienced Inventory (COPE) [38]. The COPE is a multidimensional measure that evaluates adaptive and maladaptive coping strategies, including active coping, planning, seeking instrumental social support, seeking emotional social support, suppression of competing activities, religion, positive reinterpretation, restraint coping, acceptance, focus on and venting of emotions, denial, mental disengagement, and behavioral disengagement strategies. For this study, 13 subscales of 4 items each were used, resulting in a total of 52 items. Responses are rated on a 4-point Likert scale, with higher scores indicating greater use of a given coping strategy. Motofelea et al. [39] highlighted that using a broader, more granular tool like the original COPE inventory allows clinicians to see exactly how a mother is spiraling into avoidance, giving healthcare providers a precise map to stage an early mental health intervention. Internal consistency for the overall scale was good (α = 0.73), and the internal consistency for each subscale ranges from 0.52 to 0.95, thus active coping α = 0.57, planning α = 0.73, suppression of competing activities α = 0.54, restraint coping α = 0.52, seeking instrumental social support α = 0.69, seeking emotional social support α = 0.86, positive reinterpretation α = 0.76, acceptance α = 0.73, religion α = 0.95, focus on and venting of emotions α = 0.58, denial α = 0.71, behavioral disengagement α = 0.71, and mental disengagement α = 0.61.

#### 2.2.7. Couples Satisfaction Index (CSI)

Relationship satisfaction was assessed using the 32-item Couples Satisfaction Index (CSI) [40]. The CSI is a widely used measure of couple relationship quality with excellent psychometric properties. Beaumont et al. [41] showed that the prevalence of sexual dysfunction during pregnancy is high, particularly among women followed for assisted reproductive technology. However, the quality of the couple’s relationship remains comparable between the assisted reproductive technology group and the spontaneous pregnancy group. Items are rated using Likert-type response formats, and higher scores indicate greater relationship satisfaction. Internal consistency in the present sample was excellent (α = 0.95).

Overall, Cronbach’s alpha coefficients ranged from 0.71 to 0.95, indicating acceptable to excellent internal consistency across all study measures.

### 2.3. Procedure and Ethical Considerations

The study received ethical approval from the Ethics Committee of Filantropia Clinical Hospital, Bucharest, Romania (Approval No. 4902, 30 May 2025). Participants were recruited between March and December 2025 either in person at the Filantropia Clinical Hospital of Obstetrics and Gynecology or online via Google Forms.

All participants received information regarding the study objectives, procedures, confidentiality of data, voluntary participation, and their right to withdraw at any time. Written informed consent was obtained prior to participation. Participants were also provided with the contact details of the principal investigator and could request a summary of the study findings upon completion of the research. Women interested in receiving psychological support were informed about the availability of support groups.

### 2.4. Data Analysis

Descriptive statistics were computed for all study variables and are presented as means and standard deviations (SDs). The normality of continuous variables was assessed using the Shapiro–Wilk test [42]. The test remains critical for validating parametric assumptions in structured datasets. Consistent with current practice, the Shapiro–Wilk test was used to evaluate the normality assumption before selecting the appropriate statistical analyses [43,44].

Independent-samples t tests were conducted to examine differences between women with high-risk and low-risk pregnancies. Pearson and partial correlation analyses were used to examine associations between negative pregnancy experiences, coping strategies, and indicators of emotional distress. These baseline models continue to drive network frameworks and feature screening. Schober et al. [45] emphasized the foundational status of both Pearson [46] and partial correlations for isolating linear dependencies while filtering out confounding factors in modern clinical data tracking. To control for multiple comparisons, the Holm correction [47] was applied with an alpha level of 0.05. The Holm step-down procedure remains highly relevant for error control without sacrificing massive statistical power. Holm correction maintains strict Family-Wise Error Rate (FWER) control while providing vastly superior power profiles compared to the rigid traditional Bonferroni standard in multi-comparison models [48].

Moderation analyses were conducted using regression-based approaches that included interaction terms between predictors. Regression [49] remains a dominant baseline architecture across contemporary modeling workflows. Hastie et al. [50] detail how linear and multivariable regressions serve as foundational benchmarks against which complex modern machine learning models must still be validated. Assumptions of normality, homoscedasticity, multicollinearity, and the absence of influential outliers were examined and met for all models.

Statistical analyses were performed using IBM SPSS Statistics for Windows, Version 30.0 [51], and Intellectus Statistics [52].

## 3. Results

The first hypothesis proposed that women with high-risk pregnancies would report significantly higher levels of pregnancy-related anxiety and prenatal depression compared to women with low-risk pregnancies. In addition, it was hypothesized that women with high-risk pregnancies would report lower use of socio-emotional support and mental disengagement coping strategies.

Levene’s tests [53] indicated that the assumption of homogeneity of variances was met for all variables included in the independent-samples *t* tests. Ref. [54] re-validated Levene’s test, tracking its ongoing utility and robustness against heavy-tailed, non-normal experimental allocations.

Table 2 presents the descriptive statistics for prenatal depression, pregnancy-related anxiety, socio-emotional support, and mental disengagement according to pregnancy risk status.

Table 3 summarizes the results of the independent-samples *t* tests comparing women with high-risk and low-risk pregnancies.

Regarding prenatal depression, Levene’s test was non-significant (*F* (1, 193) = 0.22, *p* = 0.640), indicating equality of variances between groups. The independent-samples *t* test revealed a statistically significant difference between women with high-risk pregnancies and those with low-risk pregnancies (*t* (193) = −2.16, *p* = 0.032, mean difference = −1.64, 95% CI [−3.14, −0.14]). Women with high-risk pregnancies reported significantly higher levels of depressive symptoms, as shown in Table 3.

For pregnancy-related anxiety, Levene’s test was also non-significant (*F* (1, 193) = 0.33, *p* = 0.566). Results indicated a significant group difference (*t* (193) = −3.57, *p* < 0.001, mean difference = −3.02, 95% CI [−4.68, −1.35]), showing that women with high-risk pregnancies experienced significantly higher levels of pregnancy-related anxiety compared to women with low-risk pregnancies.

Similarly, for socio-emotional support coping strategies, Levene’s test indicated equality of variances, (*F* (1, 193) = 3.10, *p* = 0.080). The independent-samples *t* test showed a statistically significant difference between groups (*t* (193) = 2.11, *p* = 0.036, mean difference = 0.90, 95% CI [0.06, 1.74]), indicating that women with high-risk pregnancies reported lower use of socio-emotional support strategies. To control for Type I error inflation due to multiple comparisons, the Holm correction was applied to both independent samples. All four group differences remained statistically significant after the correction: pregnancy-related anxiety (original *p* < 0.001; adjusted *p* < 0.004), mental disengagement coping (original *p* = 0.022; adjusted *p* = 0.044), prenatal depression (original *p* = 0.032; adjusted *p* = 0.064), and socio-emotional support coping (original *p* = 0.036; adjusted *p* = 0.036). All final adjusted *p*-values remained below the 0.05 threshold. Three of the four comparisons remained statistically significant after Holm correction. The difference in prenatal depression was attenuated to a marginal trend (adjusted *p* = *0*.064).

For mental disengagement coping strategies, Levene’s test was non-significant (*F* (1, 193) = 0.24, *p* = 0.628). The independent-samples *t* test revealed a statistically significant difference between women with high-risk and low-risk pregnancies (*t* (193) = 2.31, *p* = 0.022, mean difference = 0.82, 95% CI [0.12, 1.53]). Women with high-risk pregnancies reported significantly lower levels of mental disengagement coping.

Regarding Cohen’s *d* values [55] and effect-size r, for prenatal depression the values were *d* = −0.31 and r = −0.15, for pregnancy-related anxiety *d* = −0.51 and r = −0.24, for socio-emotional support *d* = 0.30 and r = 0.14, and for mental disengagement *d* = 0.33 and r = 0.16. Lakens [56] details the evolving application of Cohen’s (d) guidelines, showing how it remains central to interpreting practical significance and completing high-utility meta-analyses.

The second hypothesis proposed that negative pregnancy experiences would be positively associated with perceived stress, state anxiety (STAI), pregnancy-related anxiety (PRAS), and prenatal depression, even after controlling for pregnancy risk status.

Partial correlation analyses controlling for pregnancy risk status were conducted to examine the associations between negative pregnancy experiences and indicators of emotional distress. The Holm correction was applied to control for multiple comparisons using an alpha level of 0.05.

Results indicated that negative pregnancy experiences were significantly and positively associated with state anxiety (STAI) (r = 0.33, *p* < 0.001), pregnancy-related anxiety (PRAS) (r = 0.42, *p* < 0.001), prenatal depression (r = 0.36, *p* < 0.001), and perceived stress (r = 0.39, *p* < 0.001) after controlling for pregnancy risk status.

Additionally, negative pregnancy experiences were positively associated with perceived helplessness (r = 0.38, *p* < 0.001), as shown in Table 4.

The third hypothesis proposed that maladaptive coping strategies would be positively associated with prenatal anxiety and prenatal depression, whereas adaptive coping strategies would demonstrate differentiated associations, reflecting their role in emotional regulation.

Pearson correlation analyses were conducted to examine the associations between coping strategies and emotional distress variables. The Holm correction was applied to control for multiple comparisons using an alpha level of 0.05. Only associations that remained significant after Holm correction are reported.

Results indicated that maladaptive coping strategies were generally positively associated with emotional distress indicators. Prenatal depression was significantly and positively associated with denial (*r* = 0.39, *p* < 0.001), emotional discharge (*r* = 0.24, *p* = 0.011), mental disengagement (*r* = 0.24, *p* = 0.012), and behavioral disengagement (*r* = 0.36, *p* < 0.001). In addition, pregnancy-related anxiety was positively associated with emotional discharge (*r* = 0.24, *p* = 0.013), as shown in Table 5.

Regarding adaptive coping strategies, prenatal depression was significantly and negatively associated with planning (*r* = −0.37, *p* < 0.001), instrumental social support (*r* = −0.23, *p* = 0.012), socio-emotional support (*r* = −0.26, *p* = 0.002), positive reinterpretation (*r* = −0.52, *p* < 0.001), and acceptance (*r* = −0.30, *p* < 0.001). Furthermore, pregnancy-related anxiety was negatively associated with positive reinterpretation (*r* = −0.28, *p* < 0.001), as shown in Table 6.

The fourth hypothesis proposed that active coping would moderate the relationship between negative pregnancy experiences and perceived stress in the overall sample.

**Figure 1:**
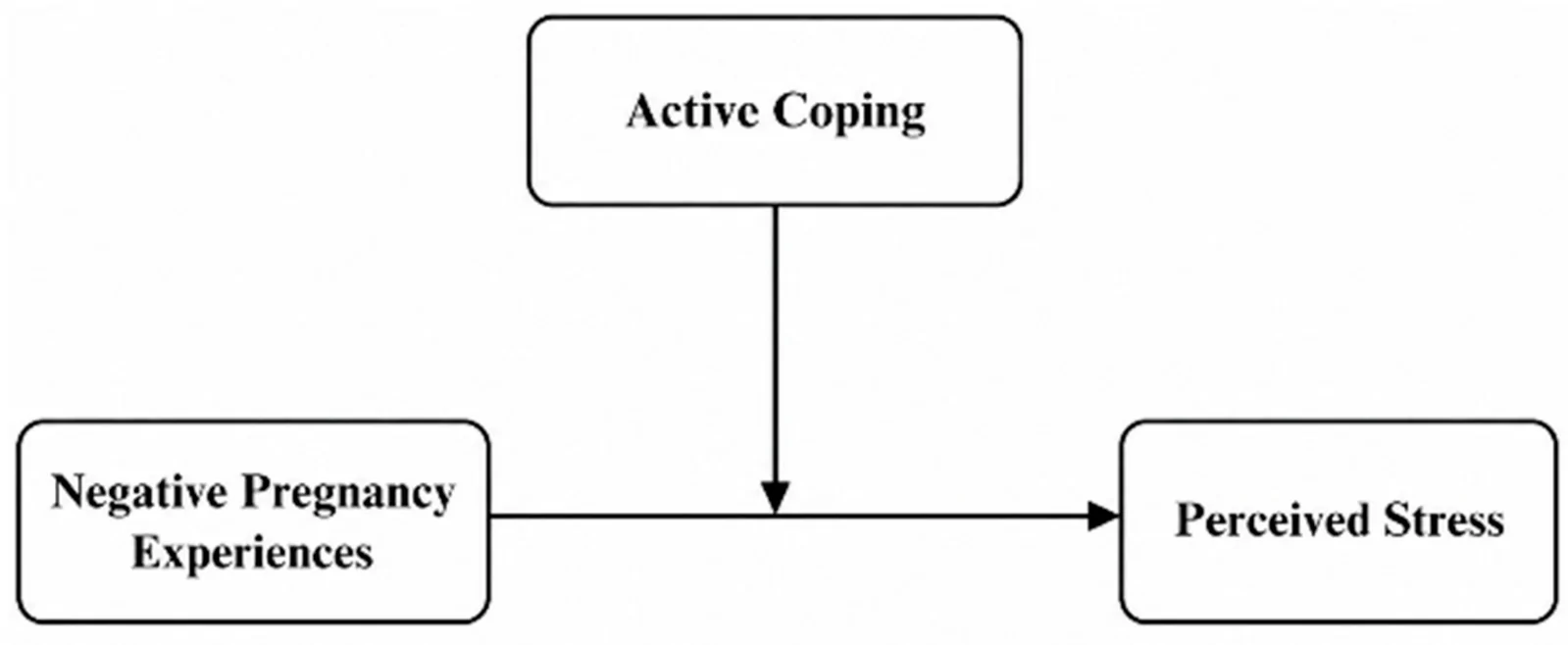
Moderating role of active coping. Source: figure created by the authors.

A moderation analysis was conducted to examine whether active coping moderated the association between negative pregnancy experiences and perceived stress (Figure 1). The overall regression model was statistically significant (*R*^2^ = 0.18, *F*(3, 191) = 14.06, *p* < 0.001), indicating that the predictors accounted for 18.09% of the variance in perceived stress.

The interaction between negative pregnancy experiences and active coping was statistically significant (*B* = −0.06, *SE* = 0.03, *β* = −0.16, *t*(191) = −2.44, *p* = 0.016), indicating a significant moderating effect. Specifically, higher levels of active coping weakened the positive association between negative pregnancy experiences and perceived stress.

The main effect of negative pregnancy experiences was significant (*B* = 0.31, *SE* = 0.05, *β* = 0.38, *t*(191) = 5.74, *p* < 0.001), suggesting that higher levels of negative pregnancy experiences were associated with increased perceived stress. In contrast, the main effect of active coping was not statistically significant (*B* = −0.03, *SE* = 0.17, *β* = −0.01, *t*(191) = −0.19, *p* = 0.846), as shown in Table 7.

**Figure 2:**
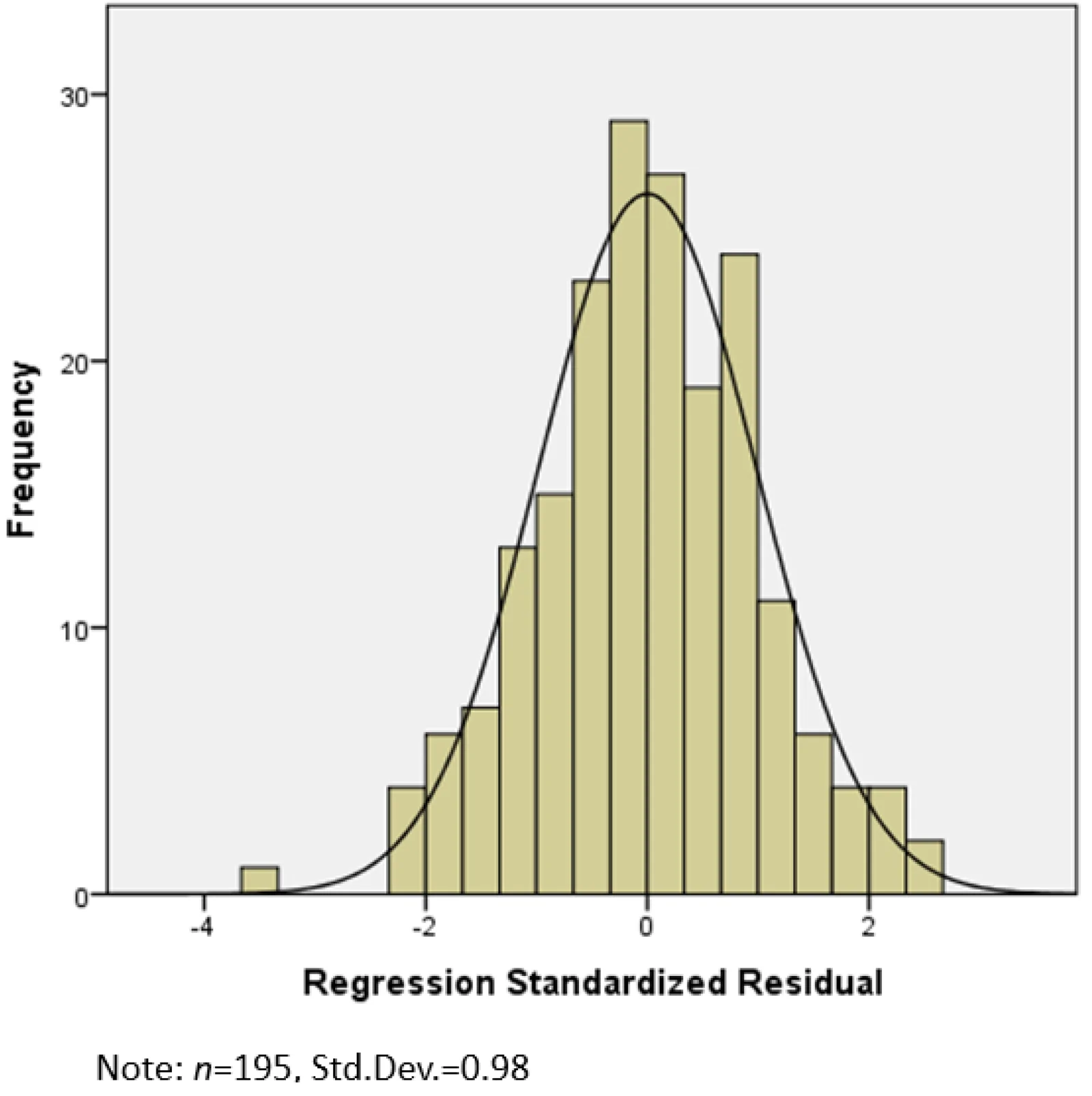
Histogram with dependent variable: perceived stress. Source: figure created in SPSS.

**Figure 3:**
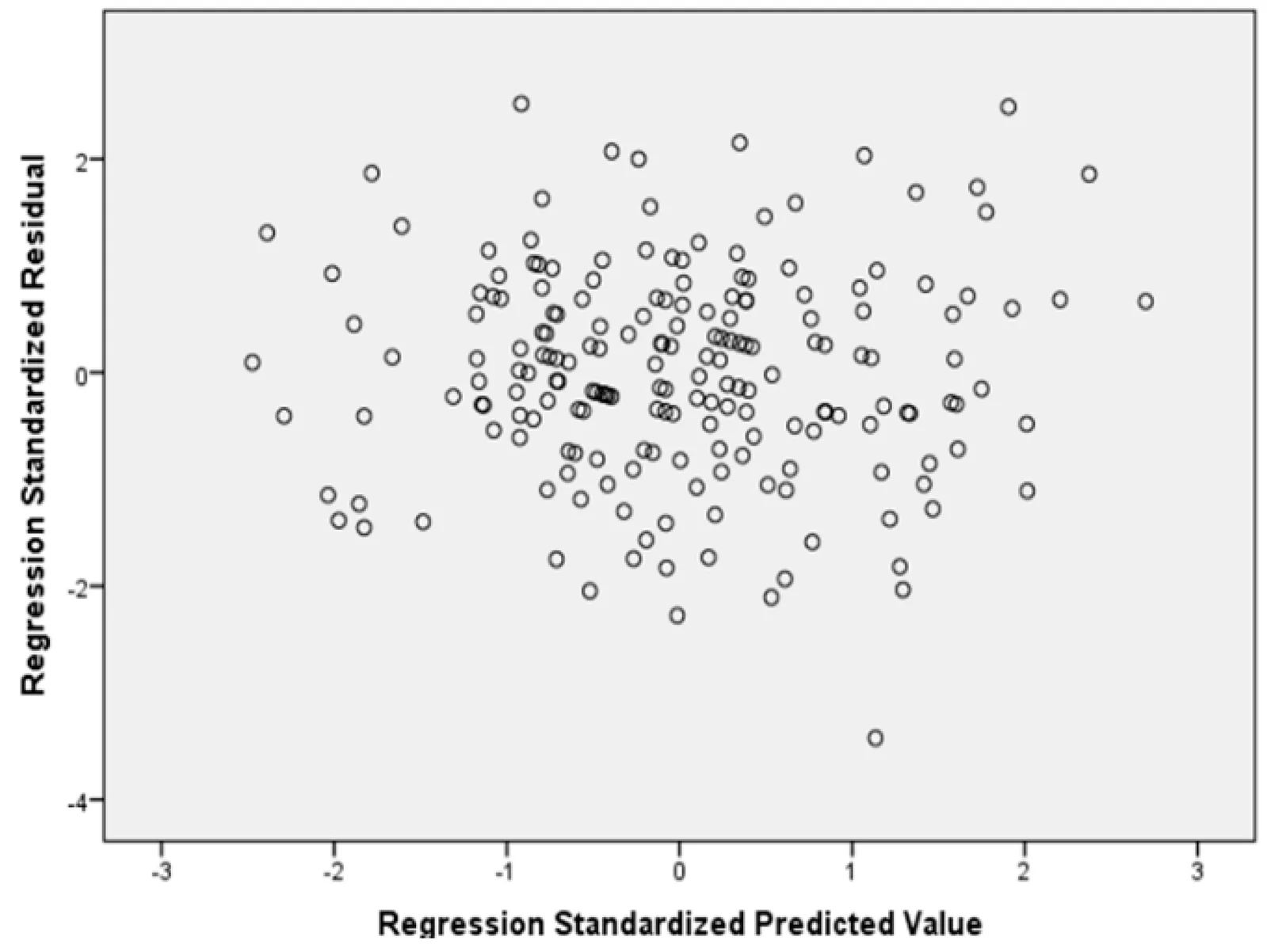
Scatterplot with dependent variable: perceived stress. Source: figure created in SPSS.

We also performed a hierarchical regression analysis for several relevant sociodemographic characteristics that revealed that negative pregnancy experiences are a robust predictor of perceived stress, accounting for 15.5% of the variance independently of sociodemographic factors. The addition of maternal age, education, socioeconomic status, and parity did not significantly alter this relationship, with negligible changes in R^2^ and no significant F-change statistics (*p* > 0.05), as shown in Table 8a,b and in Figure 2 and Figure 3.

Simple slopes analysis was conducted to further explore the effect of active coping on the relationship between negative pregnancy experiences and perceived stress. The regression coefficient for negative pregnancy experiences was calculated while holding active coping constant at its mean value, one standard deviation above the mean, and one standard deviation below the mean. The coefficient for negative pregnancy experiences with active coping fixed to a value of 10.68 was significant (B = 0.44, *p* < 0.001). The coefficient for negative pregnancy experiences with active coping fixed to a value of 12.67 was significant (B = 0.31, *p* < 0.001). The coefficient for negative pregnancy experiences with active coping fixed to a value of 14.66 was significant (B = 0.19, *p* = 0.015). This suggests that as active coping increases, the relationship between negative pregnancy experiences and perceived stress weakens. The results of the simple slopes analysis are presented in Table 9.

The fifth hypothesis proposed that marital satisfaction would moderate the relationship between prenatal depression and perceived stress among women with high-risk pregnancies.

**Figure 4:**
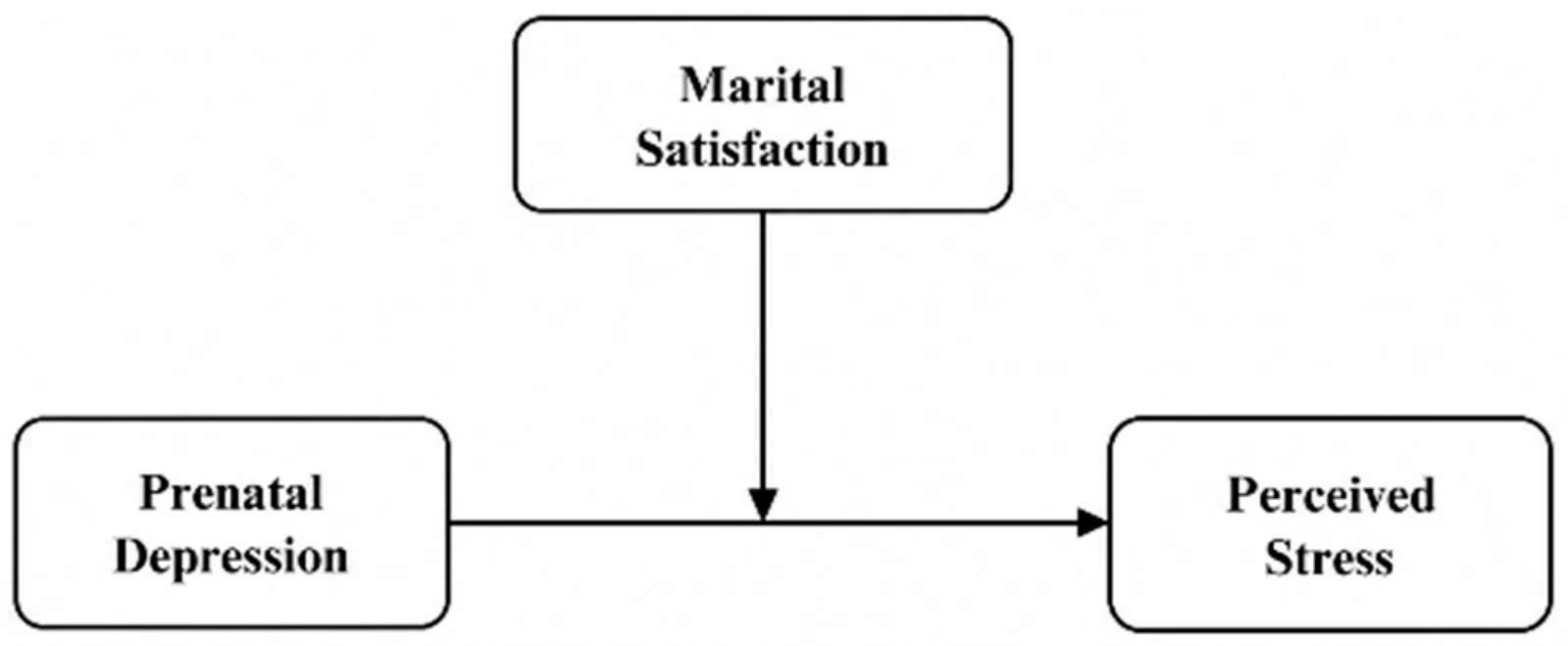
Moderating role of marital satisfaction. Source: figure created by the authors.

A moderation analysis was conducted to examine whether marital satisfaction moderated the association between prenatal depression and perceived stress in women with high-risk pregnancies (Figure 4). The overall regression model was statistically significant (*R*^2^ = 0.46, *F* (3, 98) = 27.56, *p* < 0.001), indicating that the predictors accounted for 45.76% of the variance in perceived stress.

The interaction between prenatal depression and marital satisfaction was statistically significant (*B* = −0.006, *SE* = 0.002, *β* = −0.21, *t* (98) = −2.45, *p* = 0.016), indicating a significant moderating effect. Specifically, higher levels of marital satisfaction weakened the positive association between prenatal depression and perceived stress.

The main effect of prenatal depression was significant (*B* = 0.51, *SE* = 0.08, *β* = 0.52, *t* (98) = 6.19, *p* < 0.001), suggesting that higher levels of prenatal depression were associated with increased perceived stress. In contrast, the main effect of marital satisfaction was not statistically significant (*B* = −0.02, *SE* = 0.02, *β* = −0.11, *t* (98) = −1.24, *p* = 0.219), as shown in Table 10.

Simple slopes analysis was conducted to further explore the effect of marital satisfaction on the relationship between prenatal depression and perceived stress. The regression coefficient for prenatal depression was calculated while holding marital satisfaction constant at its mean value, one standard deviation above the mean, and one standard deviation below the mean. The coefficient for prenatal depression with marital satisfaction fixed to a value of 101.17 was significant (B = 0.67, *p* < 0.001). The coefficient for prenatal depression with marital satisfaction fixed to a value of 129.45 was significant (B = 0.51, *p* < 0.001). The coefficient for prenatal depression with marital satisfaction fixed to a value of 157.73 was significant (B = 0.35, *p* = 0.002). This suggests that as marital satisfaction increases, the relationship between prenatal depression and perceived stress weakens. The results of the simple slopes analysis are presented in Table 11.

## 4. Discussion

The prenatal period represents a critical window of psychological vulnerability during which pregnant women go through risks of depression, anxiety and stress, with prevalence estimates for postnatal depression ranging from 10% to 20% globally [57,58,59]. These mental health difficulties frequently co-occur and are compounded by the demands of adjusting to the parental role, a transition that can deplete cognitive and emotional resources [59,60]. While a growing body of research has identified coping strategies, social support and marital satisfaction as factors that may mitigate perinatal distress, the specific mechanisms through which these protective resources operate, particularly their moderating functions, remain insufficiently understood [61,62].

The present study investigated the interrelationships among prenatal depressive symptomatology, anxiety, perceived stress and a comprehensive set of coping and emotion regulation strategies in a sample of perinatal women. Additionally, the study examined whether active coping and marital satisfaction functioned as moderators of the relationship between negative effects and perceived stress.

The findings revealed several noteworthy patterns. Women with high-risk pregnancies reported significantly higher levels of prenatal depression and pregnancy-related anxiety compared to women with low-risk pregnancies. This finding is consistent with previous studies suggesting that high-risk pregnancy constitutes a context of increased psychological vulnerability and emotional distress. The strong association between anxiety and depressive symptoms supports transdiagnostic models of perinatal mental health, which conceptualize anxiety and depression as overlapping manifestations of broader emotional distress rather than isolated conditions [62,63,64]. The fact that women with high-risk pregnancies use less socio-emotional support and mental passivity shows the need for further investigation of these data and the determination of causal relationships, which may include prolonged hospitalization in women with high-risk pregnancies, avoidance of negative comments, and anxiety related to uncertainty. This fact also draws attention to the need for specialized psychological assistance programs and support groups for women with high-risk pregnancies. Overall, these findings support Hypothesis 1, indicating that women with high-risk pregnancies experience higher emotional distress, reflected by elevated prenatal depression and pregnancy-related anxiety, and use less socio-emotional support and mental disengagement coping strategies.

The significant associations between negative pregnancy experiences and perceived stress, anxiety and prenatal depression suggest that the subjective appraisal of pregnancy-related difficulties plays a central role in emotional adjustment during pregnancy. These findings are consistent with cognitive models of stress and emotional vulnerability, which emphasize the importance of perceived uncontrollability and negative appraisal processes in the development of psychological distress [65,66,67]. Regarding the practical magnitude of our findings, the analysis demonstrated a medium effect in reducing pregnancy-related anxiety (d = −0.51) and small-to-medium effects on prenatal depression (d = −0.31), socio-emotional support (d = 0.30), and mental disengagement (d = 0.33). In the context of perinatal mental health, small-to-medium effect sizes are highly meaningful. Preventing or mitigating subclinical depressive and anxious symptoms during pregnancy can disrupt cumulative stress trajectories, offering substantial preventative value for both maternal psychological health and early infant development. Overall, these findings support Hypothesis 2, indicating that negative pregnancy experiences are associated with higher levels of emotional distress independently of pregnancy risk status.

Maladaptive coping strategies, particularly denial, behavioral disengagement, and mental disengagement, were positively associated with prenatal depression and anxiety. This pattern aligns with previous research showing that avoidant and disengagement coping strategies are linked with poorer psychological adjustment in perinatal populations. Moreover, the clustering of denial, mental passivity, and behavioral passivity supports theoretical frameworks describing disengagement coping as a coherent maladaptive coping dimension [3,68].

In contrast, adaptive coping strategies such as planning, acceptance, and especially positive reinterpretation were negatively associated with depressive and anxiety symptoms. Positive reinterpretation demonstrated the strongest inverse association with prenatal depression, supporting cognitive appraisal theories which propose that the ability to reframe adverse experiences in more adaptive ways buffers against emotional distress [3,69,70]. These findings suggest that cognitive flexibility and meaning-making may represent important psychological resources during pregnancy. Overall, these findings partially support Hypothesis 3, indicating that maladaptive coping strategies are associated with higher levels of emotional distress, whereas adaptive coping strategies, particularly positive reinterpretation and planning, are associated with lower levels of prenatal anxiety and depressive symptoms.

An important finding of the present study was the moderating role of active coping. Higher levels of active coping weakened the association between negative pregnancy experiences and perceived stress, indicating that active coping may function as a protective factor in the context of prenatal stress. This buffering effect is consistent with the transactional model of stress and coping [5,66], according to which active engagement with stressors reduces perceived threats and enhances feelings of control. Overall, these findings support Hypothesis 4, indicating that active coping functions as an important cognitive and behavioral buffer against the stressors tied to negative pregnancy experiences. However, it is critical to interpret the magnitude of this effect with caution. While the interaction model was statistically significant, the predictors together accounted for a relatively modest proportion of the variance in perceived stress (R^2^ = 0.18). This indicates that while active coping strategies such as planning, instrumental support seeking, and positive reframing help alter the cognitive appraisal of negative pregnancy events, they operate within a complex network of psychological, social and medical factors. The remaining 82% of unexplained variance strongly signals that active coping does not occur in a vacuum and is insufficient on its own to completely negate the heavy psychological toll of negative pregnancy experiences.

Finally, marital satisfaction buffered the relationship between prenatal depression and perceived stress among women with high-risk pregnancies. This result highlights the importance of relational resources and partner support in emotional adjustment during pregnancy and is consistent with previous findings emphasizing the protective role of supportive couple relationships in perinatal mental health [71,72,73]. This model explained a substantially larger, yet still incomplete, portion of the variance (R^2^ = 0.46). The higher explanatory power here likely reflects the acute, systemic reliance high-risk expectant mothers place on their immediate dyadic environment. Nevertheless, more than half of the variance in perceived stress for this vulnerable subgroup remains unaccounted for. Taken together, the modest-to-moderate variance explained across both models underscores that prenatal psychological distress cannot be reduced to isolated coping mechanisms or single-source interpersonal dynamics. Expectant mothers are embedded in nested systems. The etiology of prenatal stress and depression is profoundly multifactorial, driven by a complex interplay of unmeasured psychological and environmental variables. Overall, these findings support Hypothesis 5, indicating that marital satisfaction functions as a protective factor that buffers the relationship between prenatal depression and perceived stress among women with high-risk pregnancies.

Previous studies conducted in Romania have highlighted the importance of antenatal mental health screening and the identification of psychosocial risk factors associated with prenatal depression and anxiety [74].

The present findings contribute to the understanding of psychological adaptation during pregnancy by highlighting the interplay between emotional distress, coping strategies and relational resources. Beyond confirming the heightened vulnerability associated with high-risk pregnancy, the results suggest that subjective pregnancy experiences, active coping and marital satisfaction play important roles in shaping emotional adjustment. These findings support biopsychosocial models of perinatal mental health and emphasize the importance of considering both individual and interpersonal resources when examining psychological well-being during pregnancy.

### 4.1. Clinical Implications

The present findings have direct implications for prenatal care. First, women reporting frequent negative pregnancy experiences may benefit from early psychological screening and monitoring, as these experiences were consistently associated with higher levels of stress, anxiety and depressive symptoms. Second, the protective role of active coping suggests that prenatal interventions should focus on strengthening adaptive coping skills, including problem-solving, cognitive reappraisal and active engagement with pregnancy-related challenges. Our findings have clinical implications regarding the differentiation between high-risk and low-risk pregnancies. Women with high-risk pregnancies reported statistically higher levels of prenatal depression and pregnancy-related anxiety. Crucially, these observed differences extend beyond statistical significance and carry clinically meaningful differences. In psychometric and clinical terms, the score elevations observed in the high-risk group represent a shift from normative, transient situational anxiety to clinical or subclinical thresholds that warrant formal screening and targeted intervention. For these women, the psychological distress is not merely an emotional byproduct of medical complications but a distinct clinical risk factor that can negatively impact maternal adherence to medical protocols, fetal development, and obstetric outcomes.

Third, the buffering effect of marital satisfaction highlights the importance of partner involvement in perinatal care. Couple-focused interventions designed to enhance communication, emotional support, and relationship satisfaction may reduce psychological vulnerability, particularly among women facing high-risk pregnancies. Together, these findings support a biopsychosocial approach to prenatal care that addresses both medical and psychosocial determinants of maternal well-being. This recommendation is consistent with recent evidence emphasizing the value of integrating mental health assessment into routine prenatal care in order to identify women at increased psychological risk and improve maternal well-being [17,18].

When these relational and emotional support structures fail, the risk for severe perinatal mood disorders escalates significantly. In addressing these pathways, modern perinatal research emphasizes that peripartum depression remains an incredibly heterogeneous disorder dictated by a multifaceted interplay of biological, psychosocial, and environmental etiologies [75,76]. Beyond psychological distress, explicit physiological vulnerabilities can further exacerbate maternal mental decline.

For instance, recent prospective observational work has investigated physiological correlates like prepartum anemia, illustrating how closely systemic maternal health profiles can intertwine with somatic risk factors of perinatal depression [77]. Consequently, our findings support the clinical shift toward early, multi-layered perinatal screening initiatives that simultaneously evaluate individual psychological resilience, relational dyadic support, and physical risk markers to protect maternal–infant well-being.

Also, the present findings support previous recommendations advocating for the integration of mental health screening into routine prenatal care in Romania, where systematic antenatal psychological assessment remains limited [74].

The present findings are largely consistent with recent evidence published over the last five years. A systematic review by Schou Andersen et al. reported that high-risk pregnancy is consistently associated with greater psychological distress, while active coping, optimism, and empowerment represent important protective factors against adverse mental health outcomes [3]. Likewise, recent studies have confirmed that problem-focused coping is associated with lower levels of prenatal depression, anxiety, and perceived stress, whereas emotion-focused and avoidant coping strategies are linked to poorer psychological adjustment during pregnancy [5]. Our findings extend this evidence by demonstrating that active coping not only correlates with lower emotional distress but also moderates the relationship between negative pregnancy experiences and perceived stress. Furthermore, recent research has increasingly emphasized the importance of couple functioning during pregnancy. Studies involving women with high-risk pregnancies and couples experiencing gestational diabetes have shown that dyadic coping and marital satisfaction are closely associated with lower prenatal depressive symptoms and reduced stress, supporting the protective role of supportive partner relationships [78]. Collectively, these findings reinforce the view that psychological adaptation during pregnancy is determined not only by individual coping resources but also by interpersonal processes within the couple, highlighting the need for integrated psychosocial interventions during prenatal care.

### 4.2. Limitations and Future Directions

Several limitations should be acknowledged. First, the cross-sectional design of this study inherently precludes any definitive causal inference [79,80]. Future longitudinal and experimental studies are needed to determine whether active coping and marital satisfaction contribute to reductions in psychological distress over time. Second, our exclusive reliance on self-report measures introduces potential internal validity risks. This approach raises critical concerns regarding common method variance or common method bias [81,82]. Gathering data for both predictors and outcomes from the same respondents via the same questionnaire instrument can artificially inflate or deflate the observed statistical correlations.

Third, the demographic composition of our sample limits the external validity and generalizability of the findings [83,84]. The participants were predominantly from an urban setting and highly educated, a specific sub-population that may not accurately represent the broader, more diverse national or global population. Future research must utilize more inclusive sampling strategies that deliberately capture rural populations, varied socioeconomic backgrounds and diverse educational levels.

Fourth, a notable limitation of this study is the substantial amount of unexplained variance observed in our regression models, particularly within the active coping framework. Because our models focused primarily on individual coping styles and dyadic support, they did not capture broader systemic, socioeconomic, or deep-seated psychological history variables. Future research should adopt a more ecological, multi-level approach by integrating objective environmental strains (e.g., socioeconomic status, healthcare access barriers) alongside internal psychological characteristics (e.g., trait resilience, history of clinical depression) to map a more exhaustive proportion of the variance underlying prenatal distress.

Furthermore, while marital satisfaction was examined as a key moderator, the current study did not account for other contextual relationship variables, such as relationship duration, cohabitation status, history of relationship difficulties, or the psychological characteristics of the partner. The absence of these variables limits our ability to fully contextualize the dynamics of marital satisfaction in this sample. Future research should incorporate these factors to provide a more comprehensive overview of how interpersonal and history-related variables interact with the studied mechanisms.

Finally, the inherent heterogeneity within the designated high-risk group presents a confounding variable [85,86]. While participants were classified under a single high-risk umbrella, this cohort actually comprises individuals facing different types, intensities and combinations of risk factors. Future studies should employ advanced subgroup analyses, such as cluster analysis, to disentangle this heterogeneity and provide tailored insights for specific high-risk profiles. Potential confounding variables such as parity and gestational trimester were not included as covariates.

## 5. Conclusions

The present study aimed to examine the relationships among negative pregnancy experiences, emotional distress, coping strategies, and marital satisfaction during pregnancy, with particular emphasis on women with high-risk pregnancies. In addition, the study investigated whether active coping and marital satisfaction moderated the associations between negative pregnancy experiences, prenatal depression, and perceived stress.

This study highlights that emotional vulnerability during high-risk pregnancy is shaped not only by the severity of the underlying medical condition but also by the dynamic interplay between subjective pregnancy experiences, coping strategies, and relational resources. While high-risk pregnancies were associated with higher levels of prenatal depression and pregnancy-related anxiety, negative pregnancy experiences were associated with greater emotional distress, including higher levels of perceived stress, state anxiety, pregnancy-related anxiety, prenatal depression, and perceived helplessness, regardless of pregnancy risk status. Adaptive coping strategies were associated with lower levels of emotional distress, whereas maladaptive coping strategies were associated with higher levels of depressive and anxiety symptoms.

A key contribution of the study is the identification of two modifiable protective factors. Active coping attenuated the association between negative pregnancy experiences and perceived stress, whereas marital satisfaction buffered the relationship between prenatal depression and perceived stress among women with high-risk pregnancies. These findings underscore the importance of adaptive coping and relationship quality in emotional adjustment during pregnancy.

From a clinical perspective, the results support the integration of psychological screening, coping assessment, and partner involvement into routine prenatal care, particularly for women experiencing medical complications. Future longitudinal studies are needed to clarify causal pathways and evaluate interventions aimed at strengthening coping resources and relationship support during the perinatal period.

## Figures and Tables

**Table 1:** (**a**) Sociodemographic and obstetric characteristics of participants (*n* = 195). (**b**) High-risk pregnancy (*n* = 102).

**(a)**			
**Variable**	**Category**	***n***	**%**
Marital status	Married	170	87.2
	Lives with partner	21	10.8
	Other situations	4	2
Hometown	Urban	145	74.4
	Rural	50	25.6
Educational level	University studies	78	40
	Postgraduate studies	76	39
	Other forms	41	21
Professional status	Employed	167	85.6
	Housewife	15	7.7
	Self-employed	13	6.7
Economic status	Low income	10	5.1
	Average income	155	79.5
	High income	25	12.8
	No answer	5	2.6
Primiparity	Yes	121	62.1
	No	74	37.9
Pregnancy trimester	First trimester	30	15.4
	Second trimester	72	36.9
	Third trimester	93	47.7
Pregnancy risk	Low risk	93	47.7
	High risk	102	52.3
**(b)**			
**High-Risk Pregnancy**		***n***	**%**
Rh incompatibility		18	17.6
Assisted human reproduction techniques		16	15.6
Preeclampsia		12	11.7
Uterine pathology		12	11.7
Gestational diabetes		10	9.9
Infectious diseases		8	7.9
Chronic diseases		7	6.9
Overweight		7	6.9
Anemia		5	4.9
Genetic abnormalities		4	4
Placenta previa		2	2
Cholestasis of pregnancy		1	0.9

**Table 2:** Group statistics.

Std. Deviation	Mean	*n*		
5.483	7.02	93	Low-risk	Prenatal depression
5.123	8.67	102	High-risk	
6.212	20.28	93	Low-risk	Pregnancy-related anxiety
5.564	23.29	102	High-risk	
2.801	12.88	93	Low-risk	Socio-emotional support
3.131	11.98	102	High-risk	
2.577	10.01	93	Low-risk	Mental disengagement
2.399	9.19	102	High-risk	

**Table 3:** Differences between women with high-risk and low-risk pregnancies for emotional distress and coping variables.

*t*-Test for Equality of Means							Levene’s Test for Equality of Variances			
95% Confidence Interval of the Difference		Std. Error Difference	Mean Difference	Sig. (2-Tailed)	df	t	Sig.	F		
Upper	Lower									
−0.147	−3.143	0.76	−1.645	0.032	193	−2.166	0.64	0.22	Equal variances assumed	Prenatal depression
−0.142	−3.148	0.762	−1.645	0.032	188.156	−2.159			Equal variances not assumed	
−1.351	−4.678	0.843	−3.015	0	193	−3.574	0.566	0.33	Equal variances assumed	Pregnancy-related anxiety
−1.342	−4.687	0.848	−3.015	0	185.437	−3.556			Equal variances not assumed	
1.743	0.059	0.427	0.901	0.036	193	2.111	0.08	3.101	Equal variances assumed	Socio-emotional support
1.739	0.064	0.425	0.901	0.035	192.934	2.122			Equal variances not assumed	
1.527	0.122	0.356	0.824	0.022	193	2.313	0.628	0.236	Equal variances assumed	Mental disengagement
1.53	0.119	0.358	0.824	0.022	187.952	2.306			Equal variances not assumed	

**Table 4:** Partial correlations between negative pregnancy experiences and emotional distress variables controlling for pregnancy risk status.

Variable	1	2	3	4	5	6
1. Negative pregnancy experiences	-					
2. Anxiety (STAI)	0.33 *	-				
3. Pregnancy-related anxiety (PRAS)	0.42 *	0.52 *	-			
4. Prenatal depression (EPDS)	0.36 *	0.78 *	0.52 *	-		
5. Perceived stress (PSS)	0.39 *	0.60 *	0.38 *	0.56 *	-	
6. Perceived helplessness (PSS)	0.38 *	0.71 *	0.41 *	0.67 *	0.95 *	-

Note. ‘*’ indicates *p* < 0.05.

**Table 5:** Pearson correlation between maladaptive coping strategies and emotional distress variables.

Variable	1	2	3	4	5	6	7	8
1. Prenatal depression	-							
2. Pregnancy-related anxiety	0.54 *	-						
3. Suppression of competing activities	−0.12	−0.02	-					
4. Denial	0.39 *	0.17	−0.01	-				
5. Emotional discharge	0.24 *	0.24 *	0.28 *	0.20	-			
6. Mental disengagement	0.24 *	0.13	0.11	0.47 *	0.33 *	-		
7. Behavioral disengagement	0.36 *	0.20	0.09	0.49 *	0.18	0.40 *	-	
8. Restraint coping	−0.15	−0.13	0.50 *	0.21 *	0.09	0.14	0.29 *	-

Note. ‘*’ indicates *p* < 0.05.

**Table 6:** Pearson correlation between adaptive coping strategies and emotional distress variables.

Variable	1	2	3	4	5	6	7	8
1. Prenatal depression	-							
2. Pregnancy-related anxiety	0.54 *	-						
3. Planning	−0.37 *	−0.17	-					
4. Instrumental social support	−0.23 *	0.00	0.52 *	-				
5. Socio-emotional support	−0.26 *	−0.05	0.37 *	0.76 *	-			
6. Positive reinterpretation	−0.52 *	−0.28 *	0.53 *	0.45 *	0.50 *	-		
7. Acceptance	−0.30 *	−0.02	0.41 *	0.34 *	0.36 *	0.64 *	-	
8. Religious coping	−0.01	−0.15	0.04	0.11	0.21 *	0.21 *	0.16	-

Note. ‘*’ indicates *p* < 0.05.

**Table 7:** Moderation analysis predicting perceived stress from negative pregnancy experiences and active coping.

Predictor	*B*	*SE*	β	*t*	*p*
(Intercept)	19.85	0.34	0.00	58.11	<0.001
Negative pregnancy experiences	0.31	0.05	0.38	5.74	<0.001
Active coping	−0.03	0.17	−0.01	−0.19	0.846
Negative pregnancy experiences × Active coping	−0.06	0.03	−0.16	−2.44	0.016

Note. Dependent variable: perceived stress (PSS). *R*^2^ = 0.18, *F*(3, 191) = 14.06, *p* < 0.001.

**Table 8:** (**a**) Hierarchical regression analysis. (**b**) Hierarchical regression analysis.

**(a)**							
**Model**	**R**	**R Square**		**Adjusted R Square**	**Std. Error of the Estimate**	**Change Statistics**	
						**R Square Change**	**F Change**
1	0.394 ^a^	0.155		0.151	4.797	0.155	35.483
2	0.400 ^b^	0.160		0.151	4.796	0.005	1.108
3	0.404 ^c^	0.163		0.150	4.799	0.003	0.766
4	0.405 ^d^	0.164		0.146	4.811	0.000	0.067
5	0.418 ^e^	0.175		0.153	4.792	0.011	2.511
**(b)**							
**Model**		**Change Statistics**				**Durbin–Watson**	
		**df1**	**df2**		**Sig. F Change**		
1		1 ^a^	193		0.000		
2		1 ^b^	192		0.294		
3		1 ^c^	191		0.383		
4		1 ^d^	190		0.795		
5		1 ^e^	189		0.115	2.060	

^a^ Predictors: (constant), negative pregnancy experiences; ^b^ predictors: (constant), negative pregnancy experiences, maternal age; ^c^ predictors: (constant), negative pregnancy experiences, maternal age, education; ^d^ predictors: (constant), negative pregnancy experiences, maternal age, education, socioeconomic status; ^e^ predictors: (constant), negative pregnancy experiences, maternal age, education, socioeconomic status, parity; dependent variable: perceived stress.

**Table 9:** Simple slopes analysis for active coping moderating the relationship between negative pregnancy experiences and perceived stress.

Values of Active Coping	*B*	*SE*	% CI	*t*	*p*
10.68	0.44	0.07	[0.30, 0.58]	6.13	<0.001
12.67	0.31	0.05	[0.21, 0.42]	5.74	<0.001
14.66	0.19	0.08	[0.04, 0.34]	2.44	0.015

**Table 10:** Moderation analysis predicting perceived stress from prenatal depression and marital satisfaction in high-risk pregnancies.

Predictor	*B*	*SE*	β	*t*	*p*
(Intercept)	19.92	0.40	0.00	49.68	<0.001
Prenatal depression	0.51	0.08	0.52	6.19	<0.001
Marital satisfaction	−0.02	0.02	−0.11	−1.24	0.219
Prenatal depression × Marital satisfaction	−0.006	0.002	−0.21	−2.45	0.016

Note. Dependent variable: perceived stress (PSS). *R*^2^ = 0.46, *F*(3, 98) = 27.56, *p* < 0.001.

**Table 11:** Simple slopes analysis for marital satisfaction moderating the relationship between prenatal depression and perceived stress.

**Values of Marital Satisfaction**	***B***	***SE***	**% CI**	***T***	***p***
101.17	0.67	0.10	[0.47, 0.87]	6.62	<0.001
129.45	0.51	0.08	[0.34, 0.67]	6.19	<0.001
157.73	0.35	0.11	[0.13, 0.56]	3.18	0.002

## Data Availability

The original contributions presented in this study are included in the article. Further inquiries can be directed to the corresponding author.
